# University of Louisiana

**Published:** 1872-07

**Authors:** 


University of Louisiana.--We have received the announcement of the course of lectures in the Medical Department of the University of Louisiana, for the session of 1872–'73, from which we learn that Prof. Warren Stone has retired from the school, and our friend, Prof. T. G. Richardson, M.D., has been transferred to the vacant chair of surgery thus created. The vacancy created in the anatomical chair, by the transfer of Prof. Richardson, has been filled by the election of Prof. Samuel Logan, M.D. Prof. Logan is already widely and favorably known as a teacher, through his connection with the New-Orleans School of Medicine, and as a cultivated observer and accomplished writer, by his numerous contributions to the current literature of the profession.

